# Would they accept it? An interview study to identify barriers and facilitators to user acceptance of a prescribing advice service

**DOI:** 10.1186/s12913-022-07927-1

**Published:** 2022-04-18

**Authors:** Rachel Constance Yager, Natalie Taylor, Sophie Lena Stocker, Richard Osborne Day, Melissa Therese Baysari, Jane Ellen Carland

**Affiliations:** 1 Department of Clinical Pharmacology and Toxicology, St Vincent’s Hospital, 390 Victoria Street, Darlinghurst, NSW 2010 Australia; 2 School of Medical Sciences, University of NSW, Kensington, NSW Australia; 3 School of Population Health, University of NSW, Kensington, NSW Australia; 4 St Vincent’s Clinical Campus, University of NSW, Kensington, NSW Australia; 5 School of Pharmacy, Faculty of Medicine and Health, The University of Sydney, Sydney, NSW Australia; 6 Biomedical Informatics and Digital Health, School of Medical Sciences, Charles Perkins Centre, Faculty of Medicine and Health, The University of Sydney, Sydney, NSW Australia

**Keywords:** Vancomycin, Therapeutic drug monitoring, Theoretical domains framework, Consultative service, Dose prediction software

## Abstract

**Objectives:**

Few studies have explored the factors influencing user uptake of interventions designed to enhance therapeutic drug monitoring (TDM). This study aimed to identify barriers and facilitators to acceptance of a pilot intervention, the TDM Advisory Service (the Service), that provided prescribing advice for the antibiotic, vancomycin at an Australian public hospital.

**Methods:**

A sample of prescribers and pharmacists who had interacted with the Service (*n* = 10), and a sample who had not (*n* = 13), participated in semi-structured interviews. Interviews were transcribed verbatim and analysed independently by two researchers for emerging themes. The Theoretical Domains Framework (TDF) was used to synthesise barriers and facilitators to Service acceptance.

**Results:**

Key barriers reported by participants who had interacted with the Service aligned with two TDF domains: ‘Social Influences’ (prescribing hierarchy) and ‘Environmental Context and Resources’ (accessibility of dose advice). For participants who had not interacted with the Service, key barriers aligned with two TDF domains: ‘Knowledge’ (uncertainty of Service processes) and ‘Environmental Context and Resources’ (accessibility of dose advice). Key facilitators for both participant groups aligned with ‘Beliefs about Consequences’ (improved prescribing and patient outcomes) and ‘Environmental Context and Resources’ (accessibility of dose advice). A novel domain, ‘Trust’, was identified.

**Conclusions:**

Independent of participant interaction with the Service, knowledge of Service processes, perceived beneficial outcomes, improved accessibility, and trust in Service capabilities were key determinants of acceptance. This evidence can be used to inform the adoption of strategies to adapt and enhance integration of the Service into clinical workflow.

**Supplementary Information:**

The online version contains supplementary material available at 10.1186/s12913-022-07927-1.

## Background

Therapeutic drug monitoring (TDM) is the process by which biological samples (e.g. blood samples) are collected from a patient and analysed to determine the concentration of drug present to help guide subsequent dosing regimens [1]. The overall aim is to individualise drug dosing for each patient to achieve the therapeutic effect and/or avoid adverse effects. Vancomycin, a glycopeptide antibiotic, is the gold standard therapy for serious methicillin-resistant *Staphylococcus aureus* (MRSA) infections [2]. TDM is recommended for vancomycin due to its narrow therapeutic range, significant interpatient pharmacokinetic variability and associated concentration-dependent adverse effects, including nephrotoxicity [3–5]. Given that mortality from MRSA septicaemia and vancomycin-induced acute kidney injury is approximately 40 and 15%, respectively [6, 7], optimal dosing to limit the incidence of these outcomes is imperative. Despite prescriber awareness of available guidelines for vancomycin dosing and TDM [8, 9], audits of vancomycin therapy demonstrate suboptimal practices [9–11]. Of particular concern is the observed failure to adjust dosing appropriately in response to TDM [11]. Interventions designed to improve uptake of TDM-informed dose advice, including pharmacist-led initiatives [12], education programs [13] and clinical decision support systems [14], have had variable success. This variability is likely related to how readily interventions were integrated into user workflow.

Studies that have explored factors influencing uptake of TDM interventions have identified barriers including poor coordination of TDM processes, time constraints, perceived lack of user competency and the prescribing hierarchy [8, 15, 16]. While these findings are useful, systematically identifying barriers and facilitators to uptake using a theory-based framework provides an evidence-based approach to enhance intervention design and achieve successful integration into routine workflow [17, 18]. The Theoretical Domains Framework (TDF; Table 1) [19] has been successfully employed to understand barriers and facilitators to uptake of clinical interventions, such as best practices for management of nasogastric tubes [20], and to design targeted strategies to enhance implementation [20–22].

**Table 1:** The Theoretical Domains Framework with domain definitions and associated constructs

Domain (definition)	Constructs
1. Knowledge (An awareness of the existence of something)	• Knowledge (including knowledge of condition/scientific rationale) • Procedural knowledge • Knowledge of task environment
2. Skills (An ability or proficiency acquired through practice)	• Skills • Skills development • Competence • Ability • Interpersonal skills • Practice • Skill assessment
3. Social/Professional Role and Identity (A coherent set of behaviours and displayed personal qualities of an individual in a social or work setting)	• Professional identity • Professional role • Social identity • Identity • Professional boundaries • Professional confidence • Group identity • Leadership • Organisational commitment
4. Beliefs about Capabilities (Acceptance of the truth, reality or validity about an ability, talent or facility that a person can put to constructive use)	• Self-confidence • Perceived competence • Self-efficacy • Perceived behavioural control • Beliefs • Self-esteem • Empowerment • Professional confidence
5. Optimism (The confidence that things will happen for the best or that desired goals will be attained)	• Optimism • Pessimism • Unrealistic optimism • Identity
6. Beliefs about Consequences (Acceptance of the truth, reality, or validity about outcomes of a behaviour in a given situation)	• Beliefs • Outcome expectancies • Characteristics of outcome expectancies • Anticipated regret • Consequents
7. Reinforcement (Increasing the probability of a response by arranging a dependent relationship, or contingency, between the response and a given stimulus)	• Rewards (proximal/distal, valued/not valued, probable/improbable) • Incentives • Punishment • Consequents • Reinforcement • Contingencies • Sanctions
8. Intentions (A conscious decision to perform a behaviour or a resolve to act in a certain way)	• Stability of intentions • Stages of change model • Transtheoretical model and stages of change
9. Goals (Mental representations of outcomes or end states that an individual wants to achieve)	• Goals (distal/proximal) • Goal priority • Goal/target setting • Goals (autonomous/controlled) • Action planning • Implementation intention
10. Memory, Attention and Decision Processes(The ability to retain information, focus selectively on aspects of the environment and choose between two or more alternatives)	• Memory • Attention • Attention control • Decision making • Cognitive overload/tiredness
11. Environmental Context and Resources (Any circumstance of a person’s situation or environment that discourages or encourages the development of skills and abilities, independence, social competence and adaptive behaviour)	• Environmental stressors • Resources/material resources • Organisational culture/climate • Salient events/critical incidents • Person and environment interactions • Barriers and facilitators
12. Social Influences (Those interpersonal processes that can cause individuals to change their thoughts, feelings, or behaviours)	• Social pressure • Social norms • Group conformity • Social comparisons • Group norms • Social support • Power • Intergroup conflict • Alienation • Group identity • Modelling
13. Emotion (A complex reaction pattern, involving experiential, behavioural, and physiological elements, by which the individual attempts to deal with a personally significant matter or event)	• Fear • Anxiety • Affect • Stress • Depression • Positive/negative affect • Burn-out
14. Behavioural Regulation (Anything aimed at managing or changing objectively observed or measured actions)	• Self-monitoring • Breaking habit • Action planning

Adapted from Cane et al. [18]

In 2018, a TDM Advisory Service (the Service) was piloted at an Australian hospital [23]. The Service combined clinical expertise, alongside dose prediction software, to provide vancomycin dose advice to prescribers. Prior to piloting, the TDF was used to identify *anticipated* barriers to prescriber acceptance of dose prediction software recommendations [24]. Limited knowledge of software capabilities, as well as concerns about the impact on workload and patient outcomes were key barriers identified [24]. Multifaceted education strategies were implemented to address these barriers and support piloting of the Service. An interrupted time series analysis revealed that the Service increased the proportion of therapy spent in the target range [23], but provided limited information about how and why the Service was used. This current study aimed to identify barriers and facilitators to user acceptance of the Service to inform its adaptation and sustained use.

## Methods

### Study setting

Semi-structured interviews were undertaken (May 2019–January 2020) with prescribers and pharmacists to elicit their opinions of and experiences with the Service. The study was conducted at a 360-bed metropolitan Australian public hospital. An onsite pathology service provides vancomycin results 24 h-a-day, 7 days-a-week. All clinical areas, except the emergency department and outpatient clinics, use an electronic medication management system (eMMS).

### Clinical context

Prior to piloting the Service, vancomycin dosing was based on trough concentrations and was supported by internal guidelines and the Antimicrobial Stewardship (AMS) team. At the study site, most patients on vancomycin receive a consult from AMS, including review by a pharmacist and/or an ID physician. Medications are prescribed by both junior and senior prescribers in Australian hospitals, with the majority of order-entry undertaken by juniors. Pharmacists do not have prescribing rights but play an important advisory role in the prescribing process. Process maps describing vancomycin dosing and TDM processes before (Supplementary Fig. 1) and after (Supplementary Fig. 2) introduction of the Service are provided.

### TDM advisory Service

The aim of the Service was to improve compliance with antimicrobial guidelines at the institution [9] and to transition from trough- to AUC-guided dosing as per updated international consensus guidelines [25]. Service design was guided by previous research [26–31] and the expertise of a multidisciplinary committee, which included clinical pharmacologists, pathologists, infectious diseases and microbiology specialists, pharmacists, intensive care physicians and senior hospital scientists. It was tailored to accommodate local infrastructure and staffing limitations and was approved by formal governance processes.

The Service was staffed by Clinical Pharmacologists (three staff specialists and a registrar) and two senior hospital scientists. Support was provided by the AMS team. Prior to piloting, education sessions about vancomycin guidelines and Service processes were provided to all medical and surgical teams via different forums, including large scale presentations and small team meetings [23]. Attendance of education sessions was not enforced.

The Service operated on weekdays (9 am-5 pm) and provided advice after the first dose had been administered. Loading doses were prescribed with no input from the Service. Patients receiving intravenous vancomycin, regardless of infection, were identified daily from electronic prescriptions or referrals. Once identified, relevant patient information, including demographics and laboratory results, was collected and dose prediction software used to generate an individualised dose prediction to achieve an AUC_24_/MIC of 400–600 for the next 72 h of dosing. Dose predictions were generated and reviewed daily by the senior hospital scientists. After consultation with a clinical pharmacologist, dose advice was provided to clinical teams via electronic dose reports published in the patient pathology records every 72 h, or more frequently if needed. Doctors in the treating team were contacted by phone and/or page if a dose change was recommended. Acceptance of dose advice was at the discretion of the treating team.

The Service was piloted in July 2018 and transitioned to a referral basis in March 2020. Given the known lag between health system implementation and uptake, it was anticipated that not all healthcare professionals would have been aware of, or interacted directly with, the Service at the time of the study. Thus, participants were defined as either those who had not interacted (not received dose advice) with the Service, and those who had interacted (received dose advice) with the Service.

### Data collection materials

An interview guide (Supplementary File 1) was designed with input from human factors (MB), implementation (NT) and clinical pharmacology (JC, SS, RY) researchers and a clinical pharmacologist (RD). Pilot interviews were conducted with three research students and a clinical pharmacology registrar to ensure clarity.

Two process maps, describing vancomycin dosing and TDM processes before and after introduction of the Service, and an example dose report were provided to participants during interviews (Supplementary Figs. 1–3). Process maps were designed with input from clinical pharmacology researchers, a clinical pharmacologist, and the hospital TDM Committee.

### Recruitment

A targeted approach was used to recruit participants who were involved in vancomycin prescribing. Prescribers and pharmacists were invited via email or telephone to participate in the study. Initially, purposive recruitment was utilised, whereby healthcare professionals who had previous contact with the Service were invited to participate. Subsequently, a snowballing recruitment strategy was used, whereby participants recommended other colleagues involved in vancomycin prescribing. Individuals who had directly interacted with the Service, as well as those who had not interacted with the Service were recruited. Junior medical officers (JMOs), registrars, staff specialists and pharmacists were interviewed. JMOs were defined as interns (first-year postgraduate doctors) or residents (second-year postgraduate doctors). Registrars were defined as basic physician trainees or advanced trainees.

### Data collection and analysis

Interviews were conducted face-to-face or via telephone. All were audio-taped and transcribed verbatim. Identifiable data were removed from transcripts before analysis. All transcripts were analysed independently by two investigators (RY, JC). First, interviews were read to become familiar with the data. Potential themes were then identified using an inductive approach. Interviews were conducted alongside analysis to confirm thematic saturation. Finally, two investigators (RY, JC) independently synthesised barriers and facilitators to acceptance of dose advice using the TDF. Barriers and facilitators reported by participants who had not interacted with the Service, and those who had, were coded separately. Investigators met periodically to discuss findings and resolve any discrepancies. TDF coding outcomes were discussed with a third researcher (NT) when consensus could not be reached.

## Results

Interviews were conducted with prescribers (*n* = 17) and pharmacists (*n* = 6) from 25 clinical units. Some participants were affiliated with more than one team. Most participants were female (*n* = 12) and JMOs (*n* = 8). The reported experience with, and frequency of, vancomycin dosing and TDM varied (Table 2). Prescribers were reported to play key roles in the prescribing process; senior prescribers selecting the drug, while junior prescribers were responsible for “*… physically prescribing vancomycin.” (P10, JMO)*. Of those who were aware of the pilot Service (*n* = 16), 10 had received dose advice. Despite not being aware of the Service, three additional participants reported having received dose advice via telephone from the Clinical Pharmacology registrar involved in the Service. Therefore, a total of 13 participants had received dose advice from the Service. The remaining 10 participants had not received dose advice from the Service. Regardless of interactions with the Service, participants described its role as providing *“advice and information about dosing of vanc [omycin]” (P02, JMO)*. Barriers and facilitators reported by prescribers and pharmacists were similar, so are reported together.

**Table 2:** Participant demographics

Characteristic	n (%)
*Position*	
Prescriber	17 (74)
JMO	8 (35)
Registrar	7 (30)
Staff Specialist	2 (9)
Pharmacist	6 (26)
*Sex*	
Male	11 (48)
*Experience in prescribing and monitoring vancomycin*	
< 1 year	3 (13)
1–5 years	12 (52)
6–10 years	3 (13)
> 10 years	4 (17)
Unspecified	1 (4)
*Frequency of vancomycin prescribing and monitoring*	
≥ Once per day	2 (9)
≥ Once per week	3 (13)
≥ Once per month	6 (26)
≥ Once per year	6 (26)
Variable^a^	5 (22)
Unspecified	1 (4)
*Received dose advice from the Service*	
Yes	13 (57)
No	10 (43)

Percentages are rounded to whole numbers

^a^Frequency of vancomycin prescribing and monitoring varied depending on the clinical team participants were affiliated with

*JMO* Junior medical officer, *n* Number of participants

### Barriers to acceptance of the pilot Service

For participants who had not interacted with the Service, seven TDF domains encapsulated barriers to acceptance of the Service (Table 3); ‘Knowledge’ and ‘Environmental Context and Resources’ were well represented. Five TDF domains captured the barriers to acceptance of the Service reported by participants who had interacted with the Service; ‘Social Influences’, and ‘Environmental Context and Resources’ were well represented. Barriers aligned with a novel TDF domain, ‘Trust’, were identified across both groups.

**Table 3:** Reported barriers to acceptance of the pilot Therapeutic Drug Monitoring Advisory Service (the Service) aligned with the domains of the Theoretical Domains Framework (TDF)

TDF Domain	Reported barrier	Supporting participant quotes	
		Had interacted with the Service	Had not interacted with the Service
**‘Knowledge’**	Lack of procedural knowledge	*“… so I might know that actually it [trough concentration] was a peak, or actually it was taken before the wrong dose or I might just know that from being on the ward and realise that there was a mistake there, so if I’m given advice based on that mistake then I wouldn’t” (P03, JMO)*^*a*^	*“There are times when the blood is not actually taken at an appropriate time … that’s the only time I can think of, when I know there’s a problem with the test … so when there’s something I’m aware of, that the person who’s just looking at numbers on the computer is not aware of” (P13, Registrar)*^*a*^
	Lack of knowledge of the existence of the Service	*“I guess [a] major limitation would be people not knowing about it” (P06, Pharmacist)*	*“… if people are moving around from different hospitals and don’t train early on knowing that this is available...” (P09, Registrar)*
	Lack of scientific knowledge	*NR*	*“I guess the lack of evidence, again, coming back to the same point, and perhaps even clinical guidelines … some people may prefer to refer to the guidelines because a lot of work has gone into it; a lot of evidence from other sources” (P17, Pharmacist)*^*a*^
**‘Environmental Context and Resources’**	Limited accessibility of dose advice	*“The turnaround time of these reports might be a limitation … are they available when we need them?” (P21, Pharmacist)*	*“… the hours in which it runs … but it probably won’t be running on the weekend, so as long as there’s another something in place” (P04, JMO)*
	Communication issues	*NR*	*“… the only way of potentially complicating it I think is communication issues” (P09, Registrar)*
	Resources, person x environment	*NR*	*“… it depends how busy the person is, I mean if they’re on the team that has a lot of patients then electronically, they may or may not forget” (P14, JMO)*
**‘Social Influences’**	The prescribing hierarchy	*“… sometimes I’ve been asked to not chart the medication that is being prescribed, and that’s come from either ID or from one of our senior registrars and I think it’s because of the clinical picture of the patient that they’ve decided to go with the current dose” (P08, JMO)*	*“If it was a complex patient, we’d probably get an infectious diseases consult and then they could give advice as to what to do with that recommendation” (P10, JMO* ^*a*^
**‘Beliefs about Consequences’**	Belief that dose advice is incorrect	*“… perhaps if the recommended dose was very much different to what they thought it would be and what consultants might want as well, that would be a large barrier in terms of accepting and adopting that change” (P11, Pharmacist)*^*a*^	*“… if it was abnormally high or there was something that didn’t make sense. If I had seen they were previously quite stable and then it suddenly said, ‘Triple the dose’, then that would be a bit odd and you’d probably want to question.” (P20, Registrar)*^*a*^
	Increased workload	*NR*	*“… the only time I guess it could be hard to accept it is if, for example, in oncology and haematology patients when they lose weight really quickly over a week or two and that might not be updated on the [hospital electronic medication management system], so it might not represent the true weight and then you’d have to sort of manually check it to make sure that it’s all fine” (P12, JMO)*^*a*^
	Belief that dose advice will not be accessed	*“… there’s no guarantee that anyone will look at this So I suppose it’s on assumption that, you know, this information is available and prescribers and healthcare workers will look into it, or make use of it” (P19 Registrar)*	*NR*
	Concerns that dose advice is not appropriate	*NR*	*“Broadly speaking, where I would you know have concerns it’s too high or low, their renal function’s gone off since the dose recommendation had been made but I doubt there’d be too much in it” (P23, Staff Specialist)*
**‘Social/Professional Role and Identity’**	Role of senior clinicians in prescribing decision-making	*NR*	*“I could imagine sometimes, you know, the consultants in a certain setting might disagree with it” (P23, Staff Specialist)*
**‘Skills’**	Deskilling of healthcare professionals	*“I think it does take away your own learning process … I used to know by heart what the values were that I should be aiming for. Whereas now I feel like I’m becoming a bit more dependent on the system telling me what to do” (P08, JMO)*	*“I think the one [limitation] I foresee is that it will lead to a degree of deskilling, which is a problem you get with any process you introduce.” (P13, Registrar)*
**‘Memory, Attention and Decision Processes’**	Forgetting to check dose advice	*NR*	*“… you have to remember to actually look up the result online” (P12, JMO)*

^a^Quote also aligns with a novel TDF domain, ‘Trust’

*ID* Infectious diseases, *JMO* Junior medical officer, *NR* Nil report

#### Knowledge

Poor understanding of how the Service operated, including information collected and ability to cater for complex patients, was a key barrier to acceptance reported by participants who had not interacted with the Service: *“… whether or not they’re on dialysis would be a big one, but then that would have to be plugged into the software recommendation as well” (P05, Registrar)*. Lack of awareness of the existence of the Service, as well as limited scientific knowledge to interpret terminology used in dose reports, were also noted. *“I feel like I’ve prescribed vancomycin this year and this didn’t get used” (P20, Registrar)*.

#### Environmental context and resources

Accessibility of dose advice was a key concern across both groups. In particular, the lack of 24/7 decision support, dose report turnaround time and the effort required to source advice, were key barriers to acceptance of the Service. One registrar commented: *“… it’s just how accessible the information is, and how much effort I would have to go to to source the information” (P18)*.

Participants who had not interacted with the Service identified poor communication between the Service and clinical teams as a potential hinderance to acceptance: *“… with anything in healthcare, [it] comes down to how those results are communicated to a team” (P09, Registrar)*.

#### Social influences

For participants who had interacted with the Service, the prescribing hierarchy was reported to prevent acceptance. Advice provided by senior clinicians and clinical experts, including ID consultants, took precedence because *“Ultimately, they [seniors] have the final say” (P01, JMO)*.

### Facilitators to acceptance of the Service

For both participant groups two TDF domains, ‘Beliefs about Consequences’ and ‘Environmental Context and Resources’, captured key facilitators to acceptance of the Service (Table 4). Facilitators that aligned with a novel TDF domain, ‘Trust’, were also identified.

**Table 4:** Reported facilitators to acceptance of the pilot Therapeutic Drug Monitoring Advisory Service (the Service) aligned with the domains of the Theoretical Domains Framework (TDF)

TDF Domain	Reported facilitator	Supporting participant quotes	
		Had interacted with the Service	Had not interacted with the Service
**‘Beliefs about Consequences’**	Dose advice leads/will lead to better prescribing and better patient outcomes	*“… you are covered by a system that’s already been put in place to protect the patients from your lack of knowledge” (P08, JMO)*^*a*^	*“You’d probably theoretically get better control of effective levels of vancomycin and better treatment of vancomycin-required therapies” (P05, Registrar)*
	Dose advice will reduce anxiety, improve confidence, and aid prescribing decisions	*“I’m certainly imagining it will be helping prescribers and especially the junior doctors, who often aren’t confident, or not even confident with antibiotics as a whole.” (P19, Pharmacist)*	*“… it might be useful to have them [the Service] just to provide a bit of assistance in terms of dosing, in terms of therapeutic level” (P14, JMO)*
	Prescribing is/will be easier and/or more efficient with Service involvement	*“… this process seems much more efficient because we don’t really have to redo the work, we’ve already got the data there … we just thought TDM Advisory group uses that data that is available to then give a more accurate result” (P06, Pharmacist)*	*“… anything that makes our job smoother and faster people would think is positive” (P04, JMO)*
	Dose advice reduces workload	*“It saves time for prescribers in terms of dose adjustments, whether or not to review the guidelines and ‘um’ and ‘ah’ about what to do … the Service, it’s quite straightforward.” (P11, Pharmacist)*	*“I think it’ll be helpful if they’re able to order the samples for me” (P14, JMO)*
	Clinical teams will accept dose advice	*“… getting that information to them [prescribers], they will change it [the dose].” (P06, Pharmacist)*^*a*^	*“We would probably just accept it anyways” (P13, Registrar)*^*a*^
**‘Environmental Context and Resources’**	Communication	*“I feel that at least a page or a phone call to the team to notify them that this vancomycin dose is way too much for this patient or way too subtherapeutic for a patient will at least help influence the time to change the dosing” (P11, Pharmacist)*	*“… if they wanna just adjust it, that’s fine, but it would always be good to have it communicated in some way as well, either written in the notes or someone call us …*” *(P04, JMO)*
	Accessibility of dose advice	*“… having that link [the dose report], means that not only I can access it, any one of the doctors or nurses who’re involved in the patient care have access to this information because it’s there when you sign into the patient’s results …” (P19, Pharmacist)*	*“… it needs to be somewhere that’s really easily accessible, otherwise it’s gonna get lost and people won’t even realise that it’s generated” (P10, JMO)*
	Resources	*NR*	*“If it was like vastly outside of it, I’d need to look twice and I’d probably either speak to someone in clin [ical] pharm [acology] or look up like AMH or MIMS or whatever and see” (P18, Registrar)*
**‘Social Influences’**	Service operators are vancomycin prescribing experts	*“… the TDM Service are experts in therapeutic drug monitoring which is really important for vancomycin and getting the dose right..” (P03, JMO)*	*“… the way the hospital runs is different teams with different expertise provide input, so if the team with the vancomycin expertise provides input, you’d be silly to ignore it” (P10, JMO)*
	Social support	*“… we had a patient that was on vancomycin. One of the doctors asked about what dose they were on, so I said, ‘It’s been modelled by the clin [ical] pharm [acology] team and they’re on this dose and that’s giving the appropriate levels’, so I said that to reassure them that it was correct, they were happy with it” (P15, Pharmacist)*^*a*^	*“I think something that would make me take the advice on board more willingly would be talking to the microbiologist over the phone so that at least you can flag what your concerns are and they’ll take that into consideration” (P16, Registrar)*
**‘Knowledge’**	Knowledge of the existence of the Service	*“… in pharmacy we’re informed of this Service, I assume they [the Service operators] would’ve done similar education or informed the prescribers in the hospital that this is available and is of benefit and to help guide practitioners in terms of dosages. So, just getting that message, and if they are aware of that, I’m sure doctors would refer to it” (P19, Pharmacist)*	*“… they [prescribers] then need to know that this [Service] exists” (P09, Registrar)*
	Scientific knowledge	*“… if they [prescribers] understood the principles of why it’s recommended, then they would be more likely to adopt it” (P11, Pharmacist)*	*“I would take the opinion if it’s definitely evidence-based, 100% I would definitely do that. So if they [the Service operators] say that this is the recommended dose that you use for this person for this particular indication I’d definitely take that on board” (P16, Registrar)*^*a*^
	Procedural knowledge	*NR*	*“I guess it’s just people knowing that that’s a change and that that’s where it is and knowing where to find it and then maybe I guess if you do that, you don’t need to speak to infectious diseases as often” (P20, Registrar)*
**‘Beliefs about Capabilities’**	Dose advice increases/will increase professional confidence and comfort with vancomycin prescribing	*“… and that [the dose report] kind of put me at ease seeing this has been considered. The software’s saying this, look at the progress notes and they kind of tally up and I’m more comfortable with that sort of dose.” (P19, Pharmacist)*^*a*^	*“… if we’re more confident about prescribing drugs that are perceived as sort of dangerous or uncommon for us, then we would also be more comfortable and that would be a very positive thing for us” (P04, JMO)*
**‘Memory, Attention and Decision Processes’**	Understanding of vancomycin prescribing decision processes	*NR*	*“… if they just tell me “drug level is low, please adjust”, then I probably will like something like that [the dose report] that will help me decide what the next dose should be.” (P14, Registrar)*

^*a*^ Quote also aligns with a novel TDF domain, ‘Trust’

*AMH* Australian Medicines Handbook, *JMO* Junior medical officer, *MIMS* Monthly Index of Medical Specialities, *NR* Nil report

#### Beliefs about consequences

Across both groups, participants reported that acceptance of the Service was facilitated by a belief that the Service’s advice would result in better prescribing outcomes: *“… the idea is that you’re achieving better therapeutic drug monitoring and individual dosing for patients so that you can achieve therapeutic levels more efficiently and for [a] greater period of time, as well as trying to mitigate those risks of developing toxicity of vancomycin” (P02, JMO)*. Additionally, the Service was identified by both groups as resulting in increased ease and efficiency of vancomycin prescribing, including reduced workload, attributed to not needing to collect specifically timed blood samples or interpret laboratory information. One registrar noted: *“… if you don’t have to take trough levels, that’s pretty handy” (P20)*. Improved confidence and decision-making were also aligned with the dose advice.

#### Environmental context and resources

Both groups described efficient communication between the Service and medical teams, as well as easy access to online dose reports, as facilitators to acceptance of the Service. One JMO commented: *“‘What should my next dose be?’, I would make that information as easily accessible as possible so that people don’t have to rummage through” (P04)*.

### A novel TDF domain, ‘Trust’

Interview analysis revealed barriers and facilitators that aligned with a novel TDF domain, ‘Trust’ (Supplementary Table 1). Both participant groups reported trust in the Service due to the perception that those operating the Service were vancomycin prescribing experts: *“I trust the expertise of the department” (P23, Staff Specialist)*. Participants who had interacted with the Service associated the Service with *“another level of security … for the patients and for yourself” (P01, JMO)*.

A barrier to acceptance of the Service for both participant groups was a lack of trust in its ability to cater for every patient as it was *“… a bit removed” (P19, Pharmacist)* from the bedside: *“… the person who’s writing this [the dose report] probably hasn’t seen the patient in person as well, so it’d be a bit hard to trust them just based on this alone” (P16, Registrar)*. Lack of trust in the Service was particularly evident when prescribing for complex patients. A pharmacist noted: *“… it’s hard for me then to recommend it again until we work out the system for critically ill” (P21)*.

## Discussion

Limited work has been undertaken to understand user integration of TDM interventions into routine workflow [8, 15, 16]. This study applied the TDF to synthesise barriers and facilitators to user uptake of a pilot TDM Service. Key barriers and facilitators to acceptance of the Service aligned with the TDF domains of ‘Knowledge’, ‘Beliefs about Consequences’, ‘Environmental Context and Resources’, and ‘Social Influences’. A novel TDF domain, ‘Trust’, was also identified.

The TDF domains identified here are similar to those identified prior to piloting of the Service [24], but the specific barriers and facilitators that align with the domains differ. This is consistent with a recent systematic review that revealed ongoing assessment of the dynamic needs of end-users is necessary to support the design, implementation and sustainability of hospital-based interventions [32]. Before piloting, barriers aligned with ‘Knowledge’ included concerns about not having sufficient scientific knowledge to interpret dose advice [24]. In the current study, although this concern was reported by some participants who had not interacted with the Service, a dominant barrier was a lack of understanding of Service processes. Similarly, concerns about the potential negative impact of the Service on workload reported before piloting [24] were not identified in the current study. Rather, the Service was reported to have positive impacts on workload, making prescribing decisions *“so much easier” (P08, JMO).* The shift in barriers and facilitators to acceptance of the Service reinforces the value of ongoing evaluation, particularly as an intervention moves from hypothetical to actual.

Integration of the pilot Service into current vancomycin prescribing processes was welcomed by most interview participants. However, the prescribing hierarchy, as was reported by both prescribers and pharmacists, appeared to be a dominant barrier to acceptance by participants who had interacted with the Service. Although doctors are central to medication decision-making, interprofessional relationships have a strong influence on this process [33–35]. If sustained acceptance of the Service by junior doctors is to be achieved, senior prescribers must support the Service. Tailored education strategies may help overcome the apparent reluctance of some senior prescribers to accept dose advice provided by the Service [26]. Understanding why they may choose to override dose advice is also necessary. This finding emphasises that intervention design and implementation must consider all members of the clinical team, and their influence on one another.

Positive perceptions of, and trust in, clinical interventions facilitate their uptake by healthcare professionals [36, 37]. Dual coding of barriers and facilitators to ‘Trust’ and the TDF domains of ‘Social Influences’, ‘Beliefs about Consequences’, ‘Knowledge’, and ‘Beliefs about Capabilities’ highlights the complexity of trust, but also provides natural levers to enhance trust in the Service. For example, both groups of participants reported concerns about the ability of the Service to provide dose advice for complex patients (‘Beliefs about Consequences’), expressing apprehension that relevant patient characteristics were not considered. They preferred instead to consult clinical experts, such as ID consultants. ‘Knowledge’ of Service processes would have provided reassurance as an ID consultant was involved in Service operations. Understanding the capability of the Service to cater for patients in the intensive care unit [27] could also overcome these concerns. Insufficient knowledge has been reported previously to prevent uptake of clinical interventions [16]. Strategies that harness the facilitators identified in this study could build trust in the Service to support sustained uptake by end-users.

Although our study was one of the first to examine acceptance of a TDM Service in-depth, the key barriers and facilitators identified are not unique to TDM interventions. Rather, they align with those reported in work identifying factors that influence prescribing [38–41]. This finding suggests that the *end-users* and *context of use* of an intervention are critical to ensuring an intervention is accepted and used, more so than the intervention itself. Following on from this, previously reported strategies addressing contextual barriers could be employed to improve acceptance of different prescribing interventions. For example, ensuring appropriate information technology infrastructure is available at different sites is essential to minimise barriers associated with accessibility (‘Environmental Context and Resources’) and facilitate incorporation of digital interventions into the workflow of users [42, 43]. Similarly, ensuring drug-specific requirements (e.g. timing of monitoring) are known and addressed will facilitate uptake. Overall, our study indicates that understanding the specific context of the dosing decision, and addressing context-related barriers is key in supporting prescribing behaviour change.

Only two groups of healthcare professionals, prescribers and pharmacists, were interviewed. However, participants were drawn from 25 clinical units, and different levels of seniority, thereby representing a strength of the study. The TDF was not used to devise interview questions, thus interviews may not have revealed outcomes relating to all domains. However, this design ensured questions captured outcomes beyond the TDF, such as trust. Using the TDF provides a theoretical basis for the selection of strategies to support uptake of the pilot Service [17, 19, 44]. Although this study was conducted at a single-centre, and evaluated a vancomycin-targeted intervention, insights provided by the TDF may prove useful when implementing TDM interventions at other institutions.

This study showed that barriers to acceptance of a TDM advisory service included uncertainty of Service processes, the prescribing hierarchy, and potential poor accessibility of dose advice. Key facilitators included perceived improvements in prescribing and patient outcomes and easy access to the dose advice. Trust was identified as a key factor, suggesting that strategies to build trust in the Service will facilitate user acceptance. The evidence gathered will be used to inform the design and implementation of strategies to adapt and enhance integration of the Service into clinical workflow.

## Supplementary Information

**Additional file 1: Supplementary File 1.** Interview guide. **Supplementary Fig. 1.** Process map depicting vancomycin TDM processes before implementation of the pilot Service. Tools used during interviews. **Supplementary Fig. 2.** Process map depicting vancomycin TDM processes after implementation of the pilot Service. **Supplementary Fig. 3.** A dose report provided for healthcare professionals by the pilot Service. **Supplementary Table 1.** Barriers and facilitators aligned with the novel domain, Trust.

## Data Availability

The data that support the findings of this study are available from the corresponding author upon reasonable request.

## Abbreviations

AMS: Antimicrobial stewardship
ICU: Intensive care unit
ID: Infectious diseases
JMO: Junior medical officer
MRSA: Methicillin-resistant *Staphylococcus aureus*
NR: Nil report
TDF: Theoretical domains framework
TDM: Therapeutic drug monitoring
