# Supplementary material for: Would they accept it? An interview study to identify barriers and facilitators to user acceptance of a prescribing advice service
Source: BMC Health Serv Res. 2022 Apr 18;22:514. doi: 10.1186/s12913-022-07927-1 (PMC9017013; doi:10.1186/s12913-022-07927-1)
Supplement: Supplementary file 1 — Additional file 1: Supplementary File 1. Interview guide. Supplementary Fig. 1. Process map depicting vancomycin TDM processes before implementation of the pilot Service. Tools used during interviews. Supplementary Fig. 2. Process map depicting vancomycin TDM processes after implementation of the pilot Service. Supplementary Fig. 3. A dose report provided for healthcare professionals by the pilot Service. Supplementary Table 1. Barriers and facilitators aligned with the novel domain, Trust. [file 12913_2022_7927_MOESM1_ESM.docx]

**Supplementary File 1. Semi-structured interview guide**

1. Can you please start by briefly telling me about your background (e.g. pharmacy) and what your position is here at the hospital?
2. What team(s) are you currently involved with?
3. How long you have been involved in prescribing/monitoring of vancomycin?
4. How often do you prescribe/monitor vancomycin? (Prompts: how many times per day/week/month?)

*Process maps have been developed to capture how vancomycin is dosed and monitored at this hospital (show traditional process map; Supplementary Figure 1a).*

1. Does this process map accurately reflect your understanding of the process of vancomycin prescribing here at the hospital before the Service was implemented? Is there anything missing from this map?

*The TDM Advisory Service to support vancomycin dosing and monitoring was piloted from July 2018.*

1. Are you aware of the TDM Advisory Service?
   1. If no, provide interviewee with a brief background of the Service, then skip to Questions 10c, 11b, 12b and 13-19.
   2. If yes:
      - How did you find out about the Service? (Prompts: education session, colleague, contacted by the Service)
      - What team(s) were you involved with when you found out about the Service?
      - Do you know which department runs the TDM Advisory Service?
      - What is the Service?
      - How do you use the Service?
      - What is the role of the Service? How do you think the Service achieves this?
2. Has the TDM Advisory Service provided you with dose advice?
   1. If yes;
      - How did you receive the dose advice? (Prompts: phone call, dose report, another colleague)
      - How would you prefer to receive dose advice (e.g. phone, page, dose report), and why?
      - How do you think others prefer to receive dose advice? Why that method?
   2. If no, show dose report (*Supplementary Figure 1*c);
      - How do you think prescribers prefer to receive dose advice? Why this method?
3. Did you or your team accept the dose advice?
   1. What prevented/aided (or could prevent/aid) your acceptance of the dose advice?
      - If dose advice not accepted: what could help you feel more comfortable with accepting dose advice?
      - If dose advice accepted: what could prevent the acceptance of dose advice?
        - In this situation, what would help you feel more comfortable with accepting dose advice?

*We have generated a process map to outline vancomycin dosing and monitoring under the TDM Advisory Service (show Service process map; Supplementary Figure 1b).*

1. Does this process map accurately reflect how you think the Service is (or should be) involved in vancomycin prescribing and monitoring?
2. Does/would the Service impact your workflow? How?
   1. Has there been (or would there be) any change to the processes you are involved in compared to the traditional process map? (Prompt: Do/would you continue to use the guidelines? Monitor troughs?)
3. Does/would the Service impact on the workflow of your team? How?
4. What are the advantages of the Service?
5. What are the limitations of the Service?
6. Do you have any other comments/concerns regarding the Service?

Decide on appropriate drug using guidelines, senior advice

Prescribe vancomycin

Administer vancomycin

Order blood sample collection

Send to laboratory for analysis

Results received from laboratory

Identify patient

Assess clinical picture (indication, age, weight, height, etc.)

Interpret information

Collect blood sample

**1**

**2**

**10**

**3**

**4**

**5**

**9**

**6**

**7**

**8**

Supplementary Figure 1. Schematic representation of vancomycin prescribing and monitoring processes before implementation of the TDM Advisory Service.

Prescribe vancomycin

Order blood sample collection

Performed by the TDM Advisory Service

**Key**

Identify patient

Assess clinical picture (indication, age, weight, height, etc.)

Decide on appropriate drug using guidelines, senior advice

Administer vancomycin

Collect blood sample

Identify vancomycin patient

Send to laboratory for analysis

Results received from laboratory

Provide dose recommendation

TDM Advisory Service

Interpret information

**1**

**2**

**10**

**3**

**4**

**5**

**9**

**6**

**7**

**8**

**11**

**12**

**13**

Supplementary Figure 2. Schematic representation of vancomycin prescribing and monitoring processes after implementation of the pilot Therapeutic Drug Monitoring (TDM) Advisory Service.


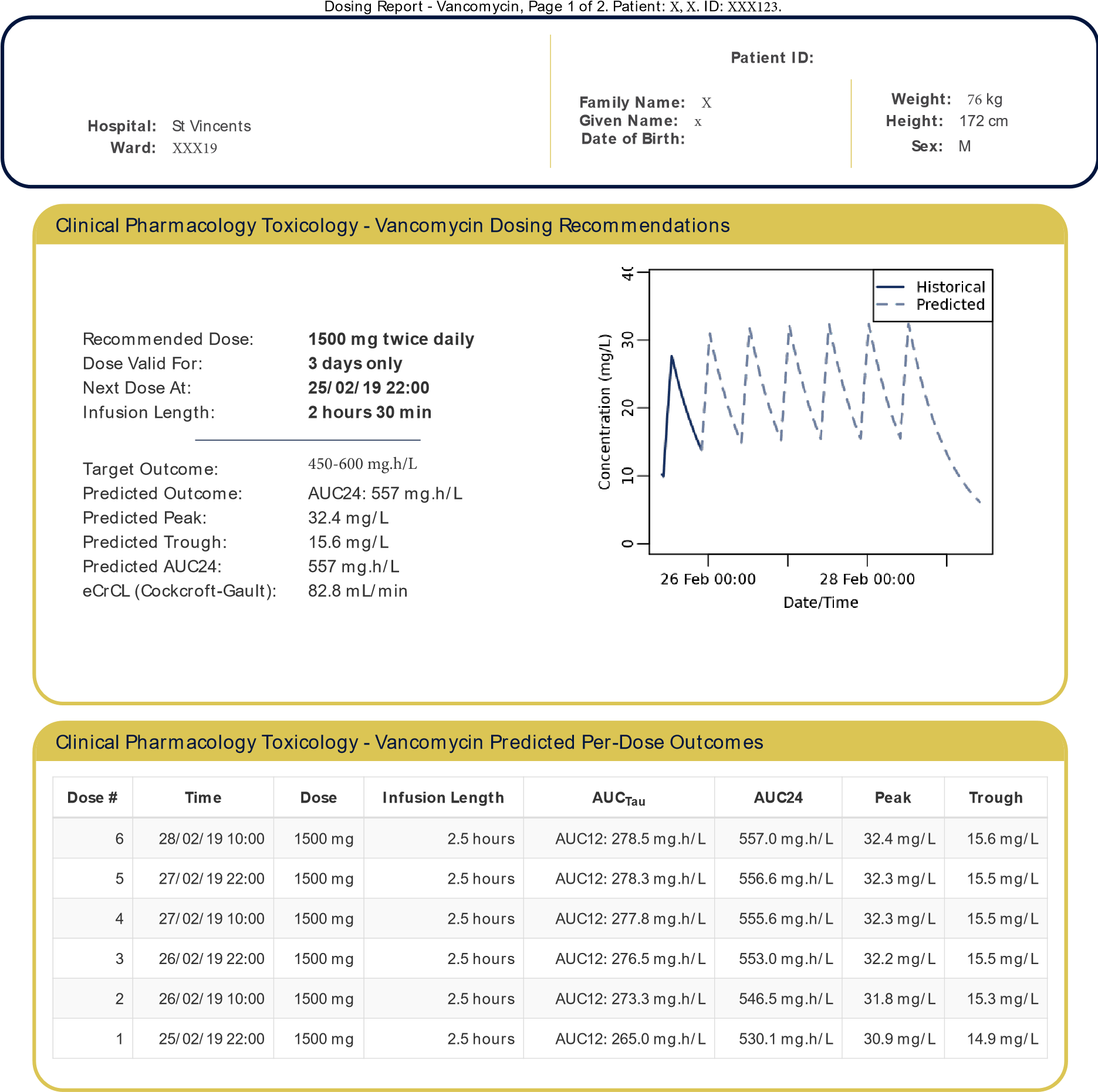


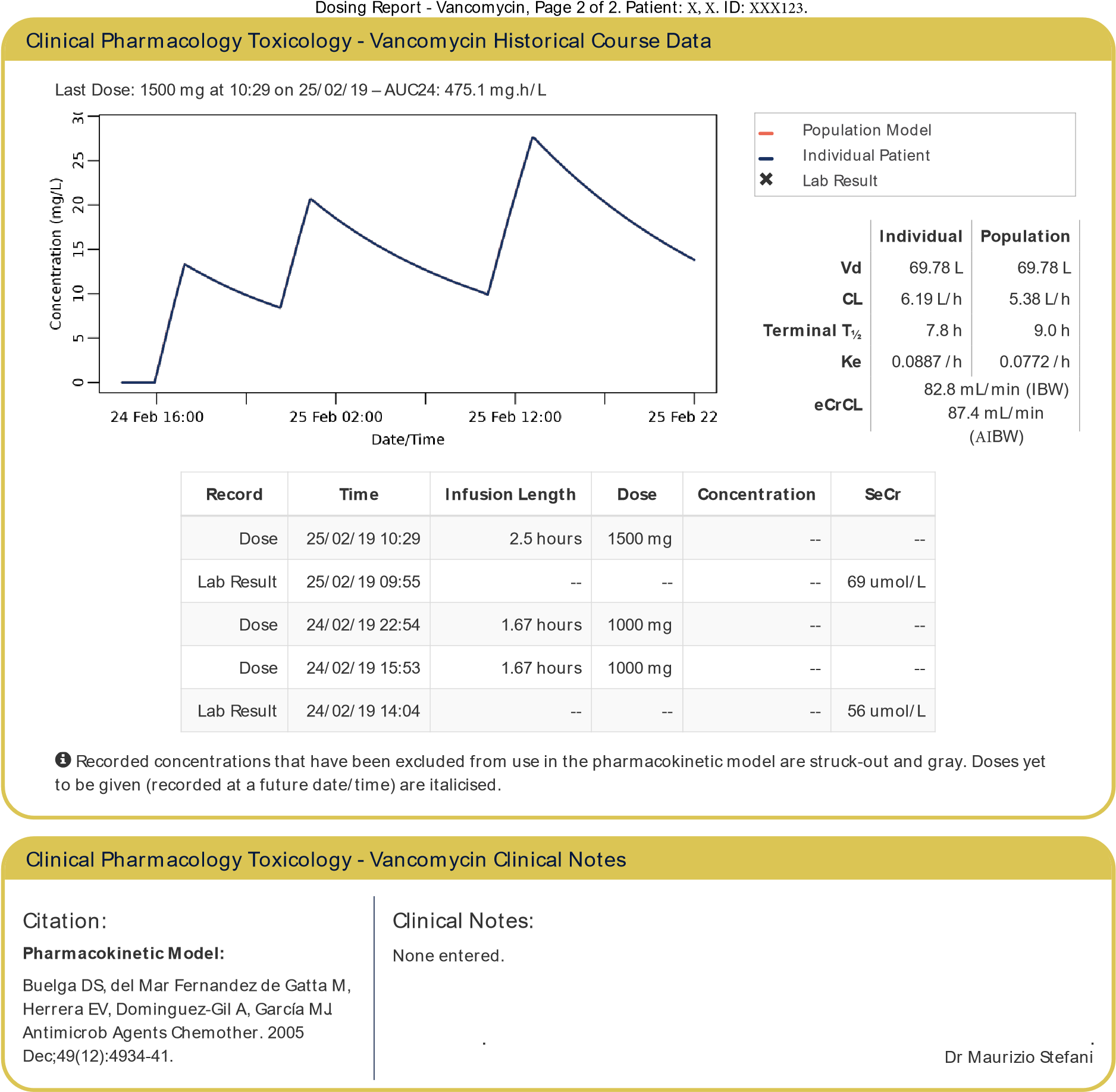


**c**

Supplementary Figure 3. An example dose report provided for healthcare professionals by the pilot Therapeutic Drug Monitoring (TDM) Advisory Service.

Supplementary Table 1. Barriers and facilitators to the acceptance of dose advice provided by the pilot TDM Advisory Service (the Service) aligned with a novel Theoretical Domains Framework (TDF) domain, ‘Trust’.

| **Reported barrier** | **Supporting quote from a participant who had interacted with the Service** | **Supporting quote from a participant who had not interacted with the Service** |
| --- | --- | --- |
| Lack of trust in capability of the Service to provide advice for complicated patients | *“…it’s hard for me then to recommend it again until we work out the system for critically ill” (P21, Pharmacist)^a^* | *“If it was a complex patient and I knew that they had some other issues, I might run it by my team or call infectious diseases or AMS.” (P10, JMO)^b^* |
| Lack of trust in capability of the Service – procedural knowledge | *NR* | *“The person who’s writing this probably hasn’t seen the patient in person as well, so it’d be a bit hard to trust them just based on this alone.” (P16, Registrar)^c^* |
| Lack of comfort with dose advice that is different to commonly prescribed doses | *“In reality, a prescriber might not be happy to make a massive leap.” (P19, Pharmacist)^a^* | *“…if it was abnormally high or there was something that didn’t make sense. If I had seen they were previously quite stable and then it suddenly said, ‘Triple the dose’, then that would be a bit odd and you’d probably want to question.” (P20, Registrar)^a^* |
| **Reported Facilitator** |  |  |
| Trust in the Service operators | *“I know that when I’m getting the advice, I know that it’s coming from the experts, so it’s always good advice.” (P03, JMO)^b^* | *“To be fair, I would probably just accept it anyways if they introduce themselves as clinical pharmacology or ID, I would just go, ‘yeah, sounds good, I will change it’.” (P13, Registrar)^b^* |
| Confidence in, and comfort provided by receipt of, dose advice provided by the Service | *“…you are covered by a system that’s already been put in place to protect the patients from your lack of knowledge” (P08, JMO)^a^* | *“A recommendation system that tells you what the dose will be would alleviate a lot of the juniors’ anxiety about it. Whether or not it’s backed up by that level of information, I don’t think will change our likelihood of accepting the information. We would probably just accept it anyways.” (P13, Registrar)^a^* |

*^a^* Quote also aligns with the TDF domain of ‘Beliefs about Consequences’.

*^b^* Quote also aligns with the TDF domain of ‘Social Influences’.

*^c^* Quote also aligns with the TDF domain of ‘Knowledge’.

AMS, antimicrobial stewardship; ICU, intensive care unit; ID, infectious diseases; JMO, junior medical officer; NR, nil report
